# Supplementary material for: No evidence of SARS-CoV-2 in hospitalized patients with severe acute respiratory syndrome in five Italian hospitals from 1st November 2019 to 29th February 2020
Source: PLoS One. 2021 Dec 7;16(12):e0260947. doi: 10.1371/journal.pone.0260947 (PMC8653811; doi:10.1371/journal.pone.0260947)

PLOS ONE

No evidence of SARS-CoV-2 in hospitalized patients with severe acute respiratory syndrome in five Italian hospitals from 1st November 2019 to 29th February 2020

--Manuscript Draft--

**Manuscript Number:**	PONE-D-21-10636
**Article Type:**	Research Article
**Full Title:**	No evidence of SARS-CoV-2 in hospitalized patients with severe acute respiratory syndrome in five Italian hospitals from 1st November 2019 to 29th February 2020
**Short Title:**	No evidence of SARS-CoV-2 in Italy from 1st November 2019 to 29th February 2020
**Corresponding Author:**	Donatella Panatto<br>University of Genoa<br>Genoa, ITALY
**Keywords:**	SARS-CoV-2 in Italy, SARI hospitalized patients, phase 1 of pandemic, influenza season 2019-2020.
**Abstract:**	Background<br>On 9 th January 2020, China CDC reported a novel coronavirus (later named SARS-CoV-2) as the causative agent of the coronavirus disease 2019 (COVID-19). Identifying the first virus introduction is of epidemiological interest for tracking and mapping SARS-CoV-2 spread in a country. In this view, a retrospective observational study was performed to detect SARS-CoV-2 in oropharyngeal samples collected from hospitalized patients with a Severe Acute Respiratory Infection (SARI) enrolled in DRIVE (Development of Robust and Innovative Vaccine Effectiveness) study in five Italian hospitals (CIRI-IT BIVE hospitals network) (1 st November 2019 – 29 th February 2020).<br>Objectives<br>To acquire new information on SARS-CoV-2 infection real trend during phase I pandemic and determine the possible virus early appearance in the Italian epidemiological scenario.<br>Materials and Methods<br>The samples were tested for SARS-CoV-2 [one-step real-time multiplex retro-transcription (RT) PCR] and for influenza [RT-PCR assay (A/H1N1, A/H3N2, B/Yam, B/Vic)] according to DRIVE study aim.<br>Results<br>Overall, 1683 samples were collected, and no evidence of SARS-CoV-2 was observed. Moreover, 28.3% (477/1683) swabs were positive for influenza. The majority were influenza type A (358 vs 119 type B). A/H3N2 was predominant among influenza A viruses (55%), and among influenza B viruses, B/Victoria was prevalent. The highest influenza incidence rate was reported in 0-17y patients (40.3%) followed by the 18-64y (24.4%) and by the ≥65y (14.8%).<br>Conclusions<br>In Italy, some studies have shown SARS-CoV-2 early circulation in northern regions, the most affected by the pandemic during phase I. On the contrary, in central and southern regions, no early circulation of the virus was registered. These results are aligned with ours. These findings enhance the need to understand the epidemiology of SARS-CoV-2 and the clinical characteristics of COVID-19 compared to other acute respiratory illnesses and evaluate the burden on the healthcare system.
**Order of Authors:**	Donatella Panatto
	Andrea Orsi
	Beatrice Marina Pennati
	Piero Luigi Lai
	Stefano Mosca
	Bianca Bruzzone
	Patrizia Caligiuri

Powered by Editorial Manager® and ProduXion Manager® from Aries Systems Corporation

	Christian Napoli
	Enrico Bertamino
	Giovanni Battista Orsi
	Ilaria Manini
	Daniela Loconsole
	Francesca Centrone
	Elisabetta Pandolfi
	Marta Luisa Ciofi Degli Atti
	Carlo Concato
	Giulia Linardos
	Andrea Onetti Muda
	Massimiliano Raponi
	Livia Piccioni
	Caterina Rizzo
	Maria Chironna
	Giancarlo Icardi
**Additional Information:**	
**Question**	**Response**
**Financial Disclosure**<br><br>Enter a financial disclosure statement that describes the sources of funding for the work included in this submission. Review the submission guidelines for detailed requirements. View published research articles from *PLOS ONE* for specific examples.<br><br>This statement is required for submission and **will appear in the published article** if the submission is accepted. Please make sure it is accurate.	The author(s) received no specific funding for this work.

Powered by Editorial Manager® and ProduXion Manager® from Aries Systems Corporation

**Unfunded studies**<br>Enter: *The author(s) received no specific funding for this work.*<br><br>**Funded studies**<br>Enter a statement with the following details:<br>• Initials of the authors who received each award<br>• Grant numbers awarded to each author<br>• The full name of each funder<br>• URL of each funder website<br>• Did the sponsors or funders play any role in the study design, data collection and analysis, decision to publish, or preparation of the manuscript?<br>• **NO** - Include this sentence at the end of your statement: *The funders had no role in study design, data collection and analysis, decision to publish, or preparation of the manuscript.*<br>• **YES** - Specify the role(s) played.<br><br>* typeset	
**Competing Interests**<br><br>Use the instructions below to enter a competing interest statement for this submission. On behalf of all authors, disclose any competing interests that could be perceived to bias this work—acknowledging all financial support and any other relevant financial or non-financial competing interests.<br><br>This statement is required for submission and **will appear in the published article** if the submission is accepted. Please make sure it is accurate and that any funding sources listed in your Funding Information later in the submission form are also declared in your Financial Disclosure statement.<br><br>View published research articles from *PLOS ONE* for specific examples.	The authors have declared that no competing interests exist.

Powered by Editorial Manager® and ProduXion Manager® from Aries Systems Corporation

**NO authors have competing interests**<br><br>Enter: *The authors have declared that no competing interests exist.*<br><br>**Authors with competing interests**<br><br>Enter competing interest details beginning with this statement:<br><br>*I have read the journal's policy and the authors of this manuscript have the following competing interests: [insert competing interests here]*<br><br>* typeset	
**Ethics Statement**<br><br>Enter an ethics statement for this submission. This statement is required if the study involved:<br><br>• Human participants<br>• Human specimens or tissue<br>• Vertebrate animals or cephalopods<br>• Vertebrate embryos or tissues<br>• Field research<br><br>Write "N/A" if the submission does not require an ethics statement.<br><br>General guidance is provided below. Consult the submission guidelines for detailed instructions. **Make sure that all information entered here is included in the Methods section of the manuscript.**	The study protocol was approved by the Ethics Committee of the Liguria Region (Genoa, Italy) (n° 245/2019).

Powered by Editorial Manager® and ProduXion Manager® from Aries Systems Corporation

**Format for specific study types**<br><br>**Human Subject Research (involving human participants and/or tissue)**<br>• Give the name of the institutional review board or ethics committee that approved the study<br>• Include the approval number and/or a statement indicating approval of this research<br>• Indicate the form of consent obtained (written/oral) or the reason that consent was not obtained (e.g. the data were analyzed anonymously)<br><br>**Animal Research (involving vertebrate animals, embryos or tissues)**<br>• Provide the name of the Institutional Animal Care and Use Committee (IACUC) or other relevant ethics board that reviewed the study protocol, and indicate whether they approved this research or granted a formal waiver of ethical approval<br>• Include an approval number if one was obtained<br>• If the study involved *non-human primates*, add *additional details* about animal welfare and steps taken to ameliorate suffering<br>• If anesthesia, euthanasia, or any kind of animal sacrifice is part of the study, include briefly which substances and/or methods were applied<br><br>**Field Research**<br><br>Include the following details if this study involves the collection of plant, animal, or other materials from a natural setting:<br>• Field permit number<br>• Name of the institution or relevant body that granted permission	
**Data Availability**<br><br>Authors are required to make all data underlying the findings described fully available, without restriction, and from the time of publication. PLOS allows rare exceptions to address legal and ethical concerns. See the PLOS Data Policy and FAQ for detailed information.	Yes - all data are fully available without restriction

Powered by Editorial Manager® and ProduXion Manager® from Aries Systems Corporation

A Data Availability Statement describing where the data can be found is required at submission. Your answers to this question constitute the Data Availability Statement and **will be published in the article**, if accepted.<br><br>**Important:** Stating 'data available on request from the author' is not sufficient. If your data are only available upon request, select 'No' for the first question and explain your exceptional situation in the text box.<br><br>Do the authors confirm that all data underlying the findings described in their manuscript are fully available without restriction?	
**Describe where the data may be found in full sentences. If you are copying our sample text, replace any instances of XXX with the appropriate details.**<br><br>• If the data are **held or will be held in a public repository**, include URLs, accession numbers or DOIs. If this information will only be available after acceptance, indicate this by ticking the box below. For example: *All XXX files are available from the XXX database (accession number(s) XXX, XXX.*).<br>• If the data are all contained **within the manuscript and/or Supporting Information files**, enter the following: *All relevant data are within the manuscript and its Supporting Information files.*<br>• If neither of these applies but you are able to provide **details of access elsewhere**, with or without limitations, please do so. For example:<br><br>*Data cannot be shared publicly because of [XXX]. Data are available from the XXX Institutional Data Access / Ethics Committee (contact via XXX) for researchers who meet the criteria for access to confidential data.*<br><br>*The data underlying the results presented in the study are available from (include the name of the third party*	All relevant data are within the manuscript and its Supporting Information files.

and contact information or URL).<br>• This text is appropriate if the data are owned by a third party and authors do not have permission to share the data.<br><br>* typeset	
Additional data availability information:	

Manuscript

Click here to access/download;Manuscript;studio DRIVE COVID_PLOSONE_31_3_21_final_1.docx

No evidence of SARS-CoV-2 in hospitalized patients with severe acute respiratory syndrome in five Italian hospitals from 1st November 2019 to 29th February 2020

Donatella Panatto[1,2], Andrea Orsi[1,2,3], Beatrice Marina Pennati[1], Piero Luigi Lai[1,2], Stefano Mosca[1], Bianca Bruzzone[3], Patrizia Caligiuri[3], Christian Napoli[4], Enrico Bertamino[5], Giovanni Battista Orsi[6], Ilaria Manini[1,7], Daniela Loconsole[8], Francesca Centrone[8], Elisabetta Pandolfi[9], Marta Luisa Ciofi Degli Atti[9], Carlo Concato[9], Giulia Linardos[9], Andrea Onetti Muda[9], Massimiliano Raponi[9], Livia Piccioni[9], Caterina Rizzo[9], Maria Chironna[8], Giancarlo Icardi[1,2,3]

[1] Interuniversity Research Center on Influenza and Other Transmissible Infections (CIRI-IT)

[2] Department of Health Sciences, University of Genoa, Italy

[3] Policlinico San Martino Hospital, Genoa, Italy

[4] Department of Medical Surgical Sciences and Translational Medicine, University La Sapienza, Italy

[5] Sant'Andrea Hospital, University La Sapienza, Italy

[6] Department of Public Health and Infectious Diseases, University La Sapienza, Italy

[7] Department of Molecular and Developmental Medicine, University of Siena, Italy

[8] Hygiene Section, Department of Biomedical Sciences and Human Oncology, University of Bari, Italy

[9] Bambino Gesù Children's Hospital, Italy

Corresponding author: Prof. Donatella Panatto

Corresponding author email: panatto@unige.it

Abstract

Background

On 9th January 2020, China CDC reported a novel coronavirus (later named SARS-CoV-2) as the causative agent of the coronavirus disease 2019 (COVID-19).

Identifying the first virus introduction is of epidemiological interest for tracking and mapping SARS-CoV-2 spread in a country. In this view, a retrospective observational study was performed to detect SARS-CoV-2 in oropharyngeal samples collected from hospitalized patients with a Severe Acute Respiratory Infection (SARI) enrolled in DRIVE (Development of Robust and Innovative Vaccine Effectiveness) study in five Italian hospitals (CIRI-IT BIVE hospitals network) (1st November 2019 – 29th February 2020).

Objectives

To acquire new information on SARS-CoV-2 infection real trend during phase I pandemic and determine the possible virus early appearance in the Italian epidemiological scenario.

Materials and Methods

The samples were tested for SARS-CoV-2 [one-step real-time multiplex retro-transcription (RT) PCR] and for influenza [RT-PCR assay (A/H1N1, A/H3N2, B/Yam, B/Vic)] according to DRIVE study aim.

Results

Overall, 1683 samples were collected, and no evidence of SARS-CoV-2 was observed. Moreover, 28.3% (477/1683) swabs were positive for influenza. The majority were influenza type A (358 vs 119 type B). A/H3N2 was predominant among influenza A viruses (55%), and among influenza B viruses, B/Victoria was prevalent. The highest influenza incidence rate was reported in 0-17y patients (40.3%) followed by the 18-64y (24.4%) and by the ≥65y (14.8%).

Conclusions

In Italy, some studies have shown SARS-CoV-2 early circulation in northern regions, the most affected by the pandemic during phase I. On the contrary, in central and southern regions, no early circulation of the virus was registered. These results are aligned with ours. These findings enhance the need to understand the epidemiology of SARS-CoV-2 and the clinical characteristics of COVID-19 compared to other acute respiratory illnesses and evaluate the burden on the healthcare system.

Introduction

On December 31, 2019, the Wuhan Municipal Health Commission in Wuhan City, Hubei province, China, reported a cluster of 27 pneumonia cases of unknown aetiology [1]. On January 9, 2020, the China CDC said that a novel coronavirus (later named SARS-CoV-2, the virus causing COVID-19) had been detected as the causative agent for 15 of the 59 cases of pneumonia [2,3].

The first European case was officially reported from France on January 24, 2020 [4, 5]. One week later, in Italy, the first cases were described. Two Chinese tourists from Wuhan landed in Milan and then fell ill in Rome on January 30, 2020 [6]. The first autochthonous patient, a 38-year-old man, was diagnosed only one month later in Codogno (Lombardy), on February 21. It was believed to be the "patient zero", but the first introduction of the virus into Italy remains unclear.

Many European countries have been trying to ascertain whether SARS-CoV-2 infections had occurred before the official 1st case reported by health authorities [7-13]. In this regard, we conducted a retrospective observational study to detect SARS-CoV-2 in oropharyngeal samples collected from hospitalized patients with severe acute respiratory infection (SARI) [14] aged ≥6 months in five hospitals in four Italian cities (Genoa, Rome, Bari, Siena) in the period 1st November 2019 – 29th February 2020. Our intention was to acquire new information on the real trend of the infection during phase I of the epidemic, and to determine the possible early appearance of the virus in Italy.

Materials and Methods

Study population and period

Samples were collected during the 2019-2020 influenza season by hospitalized individuals with SARI enrolled in the European study DRIVE (Development of Robust and Innovative Vaccine Effectiveness) [15] between 1st November 2019 and 29th February 2020.

SARI and COVID-19 definition

According to the ECDC case definition, a case of SARI is defined as a hospitalized patient of any age with at least one respiratory sign or symptom (cough, sore throat, breathing difficulties) and at least one systemic sign or symptom (fever or low-grade fever, headache, myalgia, generalized malaise) or deterioration in general condition (asthenia, weight loss, anorexia or confusion and dizziness) [15].

A case of COVID-19 is considered any person with at least one symptom such as cough, fever, shortness of breath, sudden onset of anosmia, ageusia or dysgeusia. Additional less specific symptoms may include headache, chills, muscle pain, fatigue, vomiting and/or diarrhoea [15].

Molecular analysis for SARS-CoV-2 detection

Total RNA was extracted and tested for the identification of SARS-COV-2 by means of a one-step real-time multiplex retro-transcription (RT) PCR assay targeting the nucleoprotein region (N), RNA-dependent RNA-polymerase region (RdRp) and the envelope region (E), in accordance with international protocols [16].

Molecular analysis for influenza detection

Total RNA was extracted and tested for the identification of influenza viruses (A/H1N1, A/H3N2, B/Yamagata, B/Victoria) by means of a one-step real-time multiplex retro-transcription (RT) PCR assay, in accordance with international protocols [16].

Results

Overall, 1,683 samples were collected at different times during the study period (Figure 1).

Fig.1 Number of enrollments (= swabs) distribution by week, during the study period.

Data on demographic characteristics, chronic conditions, risk factors and influenza vaccination status were available for every patient (Table 1). The patients' mean age was 38.2 years and about 35.7% (600/1,683) were ≥65 years old.

Table 1. Patients' characteristics stratified by age.

	<18 y	18-64 y	≥65 y
Total	780 (100%)	303 (100%)	600 (100%)
Sex=male	422 (54.1%)	175 (57.8%)	331 (55.2%)
**Any chronic condition***			
No (0)	666 (85.4%)	174 (57.4%)	95 (15.8%)
Yes (≥1)	114 (14.6%)	129 (42.6%)	505 (84.2%)
**Influenza vaccination status (2019-2020 season)**			
No	751 (96.3%)	265 (87.5%)	345 (57.5%)
Yes	27 (3.5%)	37 (12.2%)	254 (42.3%)
N/A	2 (0.2%)	1 (0.3%)	1 (0.2%)

* Chronic respiratory diseases, Heart or cardiovascular disease, Diabetes, Renal disease, Anemia, Cancer, Chronic liver disease, Dementia, History of stroke, Obesity, Autoimmune disease, Rheumatological diseases.

Clinical manifestations were also recorded for every patient (Table 2). The most common symptoms were fever (81.1%, 1,365/1683) and cough (60.1%, 1,012/1683) (Table 2); these are generic symptoms that could hypothetically be related to SARS-CoV-2 infection.

Table 2. Patients' clinical manifestations.

**Symptoms**	
Fever	1,365 (81.1%)
Malaise	798 (47.4%)
Headache	196 (11.6%)
Myalgia	271 (16.1%)
Cough	1,012 (60.1%)
Sore throat	731 (43.4%)
Short breath	590 (35.1%)

No evidence of SARS-CoV-2 was found.

According to DRIVE objectives we tested all swabs for influenza viruses. Generally, 28.3% (477/1,683) of our swabs were positive for influenza: 358 (75%, 358/477) were type A and 119 (25%, 119/477) type B. The details are shown in Figure 2. Most influenza cases were of subtype A/H3N2 and mainly affected subjects

aged <18 years and the elderly. By contrast, subtype A/H1N1 was prevalent in adults (aged 18-64 years), followed by the elderly (Figure 2). Subjects vaccinated against influenza in the 2019-2020 season were 18.9% (318/1,683). In particular, 79.9% (254/318) were the elderly, 11.6% (37/318) aged 18-64 years and 8.5% (27/318) aged <18 years.

Fig.2 Sample distribution by influenza type/subtype or lineage and age during the study period.

Discussion

In Italy, the first autochthonous case was diagnosed one month later in Codogno (Lombardy), on February 21, 2020. Over the following days, cases were reported from several different areas of the country, with Northern Italy being most severely affected at the beginning of the COVID-19 pandemic [17]. Identifying the first introduction of the virus is of epidemiological interest to track and map the spread of SARS-CoV-2 in a country.

The clinical information we collected showed that the definition of COVID-19 overlaps with that of SARI, confirming that the clinical picture is insufficient to diagnose SARS-CoV-2 infection [18,19]. Moreover, considering the evidence in the literature [18,19], we analyzed samples collected from hospitalized patients with SARI, as it has been demonstrated that patients with COVID-19 are more likely to be admitted to hospital; therefore, these patients were best suited to the aim of the study.

No evidence of SARS-CoV-2 was found. Our results are in line with some Italian and European data. Capalbo et al. [13] evaluated the prevalence of SARS-CoV-2 infection among SARI patients in a hospital in Central Italy from November 1, 2019 to March 1, 2020. Like us, they confirmed that SARS-CoV-2 was not circulating at the time of their study and that the COVID-19 pandemic did not start before its official onset in Italy. Similarly, in Scotland, Tomb et al. detected no SARS-CoV-2 in the population prior to March 2020 [12].

By contrast, other studies revealed the presence of the virus prior to the first “official” case. In Italy, some retrospective studies conducted on samples of a different sort (serological and environmental samples) and from patients with different health conditions reported different results from ours [7-10]. In Northern Italy, environmental wastewater monitoring detected positive samples as early as December 2019 [9,10]. In Milan, Amendola et al. detected the RNA of the virus in early December 2019 in a swab sample from a child with suspected measles [8]. Finally, Apolone et al. found seroprevalence evidence of SARS-CoV-2 in asymptomatic

patients. These were lifelong smokers who were screened for the early detection of lung cancer (high-risk group) from September to October 2019 [7].

It should be noted that the published Italian studies reporting early SARS-CoV-2 circulation were conducted in geographic areas that were different from ours, and which were severely affected during the initial phase I. In France, Deslandes et al. retrospectively analyzed nasopharyngeal swabs collected from hospitalized patients; their results suggested that the epidemic had probably started in early December 2019 [11].

The COVID-19 pandemic impacted influenza circulation from week 13 of 2020, when countries implemented strict lockdowns and issued hygiene recommendations [20]. In line with the European trend, the 2019-2020 influenza season in Italy had a shorter overall duration than previous seasons.

Like all the literature studies considered, ours has some limitations. We used oropharyngeal swabs, while it has been shown that nasopharyngeal swabs are the most suitable for the molecular detection of SARS-CoV-2, as the quantity of virus is greater in the nose [21]. Moreover, we stocked the swab aliquots at -20°C and later processed them for SARS-CoV-2 molecular research, and it is known that the defrosting step can affect the results of the extraction and real-time steps.

Conclusions

The results published in the literature show that it is very difficult to establish the exact time and place of the initial SARS-CoV-2 outbreak in Italy and Europe. These findings highlight the need to continue to carry out retrospective studies in order to understand the epidemiology of the novel coronavirus, to identify the clinical characteristics of COVID-19 in comparison with other acute respiratory illnesses (ARI), and to evaluate the burden of COVID-19 on the healthcare system.

In sum, it is crucial to strengthen routine monitoring (both epidemiological and laboratory) of the causative agents of SARI, in order to support preventive strategies for all respiratory pathogens and promote integrated strategies for influenza and COVID-19 vaccination.

Acknowledgments

All authors have read and agree to the published version of the manuscript. Conceptualization: G.I., D.P., A.O., C.N., I.M., C.R., M.C; data collection: B.M.P., P.L.L., E.B., D.L., E.P., M.L.C.D.A., M.R., C.R.; laboratory analyses: B.M.P., B.B., P.C., I.M., E.B., F.C., D.L., C.C., A.O.M, G.L.; interpretation of the data: P.L.L., A.O., D.P., G.B.O., M.C.; software, S.M.; drafting of the paper: D.P., B.M.P., A.O.; critical revision of manuscript: C.R., M.C., I.M., C.N., G.I., supervision, G.I.

References

1) Wuhan City Health Committee (WCHC). Wuhan Municipal Health and Health Commission's briefing on the current pneumonia epidemic situation in our city 2019. http://wjw.wuhan.gov.cn/front/web/showDetail/2019123108989. (Accessed on 26/03/2021)
2) News X. Experts claim that a new coronavirus is identified in Wuhan 2020. http://www.xinhuanet.com/2020-01/09/c_1125438971.htm. (Accessed on 26/03/2021)
3) Wang C, Horby PW, Hayden FG, Gao GF. A novel coronavirus outbreak of global health concern. Lancet. 2020 Feb 15;395(10223):470-473. doi: 10.1016/S0140-6736(20)30185-9. Epub 2020 January 24. Erratum in: Lancet. 2020 Jan 29; PMID: 31986257; PMCID: PMC7135038.
4) ECDC. Event background COVID-19. https://www.ecdc.europa.eu/en/novel-coronavirus/event-background-2019. (Accessed on 26/03/2021)
5) Lescure FX, Bouadma L, Nguyen D, Parisey M, Wicky PH, Behillil S, et al. Clinical and virological data of the first cases of COVID-19 in Europe: a case series. Lancet Infect Dis 2020 March 27 [Epub ahead of print]. doi:10.1016/S1473-3099(20)30200-0.
6) Giovanetti M, Benvenuto D, Angeletti S, Ciccozzi M. The first two cases of 2019-nCoV in Italy: where they come from? J. Med. Virol. 92 (5), 518–521. https://doi.org/10.1002/jmv.25699
7) Apolone G, Montomoli E, Manenti A, Boeri M, Sabia F, Hyseni I, et al. Unexpected detection of SARS-CoV-2 antibodies in the prepandemic period in Italy. Tumori. 2020 Nov 11:300891620974755. doi: 10.1177/0300891620974755. Epub ahead of print. PMID: 33176598.
8) Amendola A, Bianchi S, Gori M, Colzani D, Canuti M, Borghi E, et al. Evidence of SARS-CoV-2 RNA in an Oropharyngeal Swab Specimen, Milan, Italy, Early December 2019. Emerg Infect Dis. 2021 Feb;27(2):648-650. doi: 10.3201/eid2702.204632. Epub 2020 December 8. PMID: 33292923.

9) Bar-Or I, Yaniv K, Shagan M, Ozer E, Erster O, Mendelson E, et al. Regressing SARS-CoV-2 sewage measurements onto COVID-19 burden in the population: a proof-of-concept for quantitative environmental surveillance. medRxiv https://doi.org/10.1101/ 2020.04.26.20073569 preprint.

10) La Rosa G, Mancini P, Bonanno Ferraro G, Veneri C, Iaconelli M, Bonadonna L, et al. SARS-CoV-2 has been circulating in northern Italy since December 2019: Evidence from environmental monitoring. Sci Total Environ. 2020 August 15;750:141711. doi: 10.1016/j.scitotenv.2020.141711. Epub ahead of print. PMID: 32835962; PMCID: PMC7428442.

11) Deslandes A, Berti V, Tandjaoui-Lambotte Y, Alloui C, Carbonnelle E, Zahar JR, et al. SARS-CoV-2 was already spreading in France in late December 2019. Int. J. Antimicrob. Agents 55, 106006.

12) Tomb RM, MacLean AR, Gunson RN. Retrospective screening for SARS-CoV-2 in Greater Glasgow and Clyde ICUs between December 2019 and February 2020. J Infect. 2020 Sep;81(3):452-482. doi: 10.1016/j.jinf.2020.06.022. Epub 2020 Jun 15. PMID: 32553840; PMCID: PMC7295493.

13) Capalbo C, Bertamino E, Zerbetto A, Santino I, Petrucca A, Mancini R, et al. No Evidence of SARS-CoV-2 Circulation in Rome (Italy) during the Pre-Pandemic Period: Results of a Retrospective Surveillance. Int J Environ Res Public Health. 2020 Nov 16;17(22):8461. doi: 10.3390/ijerph17228461. PMID: 33207548; PMCID: PMC7696939

14) ILI, SARI and COVID-19 ECDC definition. Available at: https://www.ecdc.europa.eu/(Accessed on 26/03/2021)

15) DRIVE (Development of Robust and Innovative Vaccine Effectiveness). https://www.drive-eu.org/ (Accessed on 26/03/2021)

16) WHO Global Influenza Surveillance Network. Manual for the laboratory Diagnosis and Virological Surveillance of Influenza; Available online: http://whqlibdoc.who.int/publications/2011/9789241548090_eng (Accessed on 26/03/2021)

17) Epicentro, Istituto Superiore di Sanità, Italy. COVID-19 integrated surveillance data in Italy. Available from: https://www. epicentro.iss.it/en/coronavirus/sars-cov-2-dashboard. (Accessed on 26/03/2021)

18) Zheng X., Wang H., Su Z. Co-infection of SARS-CoV-2 and influenza virus in early stage of the COVID-19 epidemic in Wuhan. China J Infect. 2020;81(2):e128–e129. doi: 10.1016/j.jinf.2020.05.041.

19) Faury H, Courboulès C, Payen M, Jary A, Hausfater P, Luyt C, et al. Medical features of COVID-19 and

influenza infection: A comparative study in Paris, France. J Infect. 2020 Aug 14:S0163-4453(20)30551-X. doi: 10.1016/j.jinf.2020.08.017. Epub ahead of print. PMID: 32798533; PMCID: PMC7426213.

20) COVID-19 situation in Italy by Protezione Civile. Available at: https://gisanddata.maps.arcgis.com/apps/opsdashboard/index.html#/b0c68bce2cce478eaac82fe38d4138b1 (Accessed on 26/03/2021)

21) Wang X, Tan L, Wang X, Liu W, Lu Y, Cheng L, et al. Comparison of nasopharyngeal and oropharyngeal swabs for SARS-CoV-2 detection in 353 patients received tests with both specimens simultaneously. Int J Infect Dis. 2020 May;94:107-109. doi: 10.1016/j.ijid.2020.04.023. Epub 2020 April 18. PMID: 32315809; PMCID: PMC7166099

Figure 1

Click here to access/download;Figure;Fig1.tiff

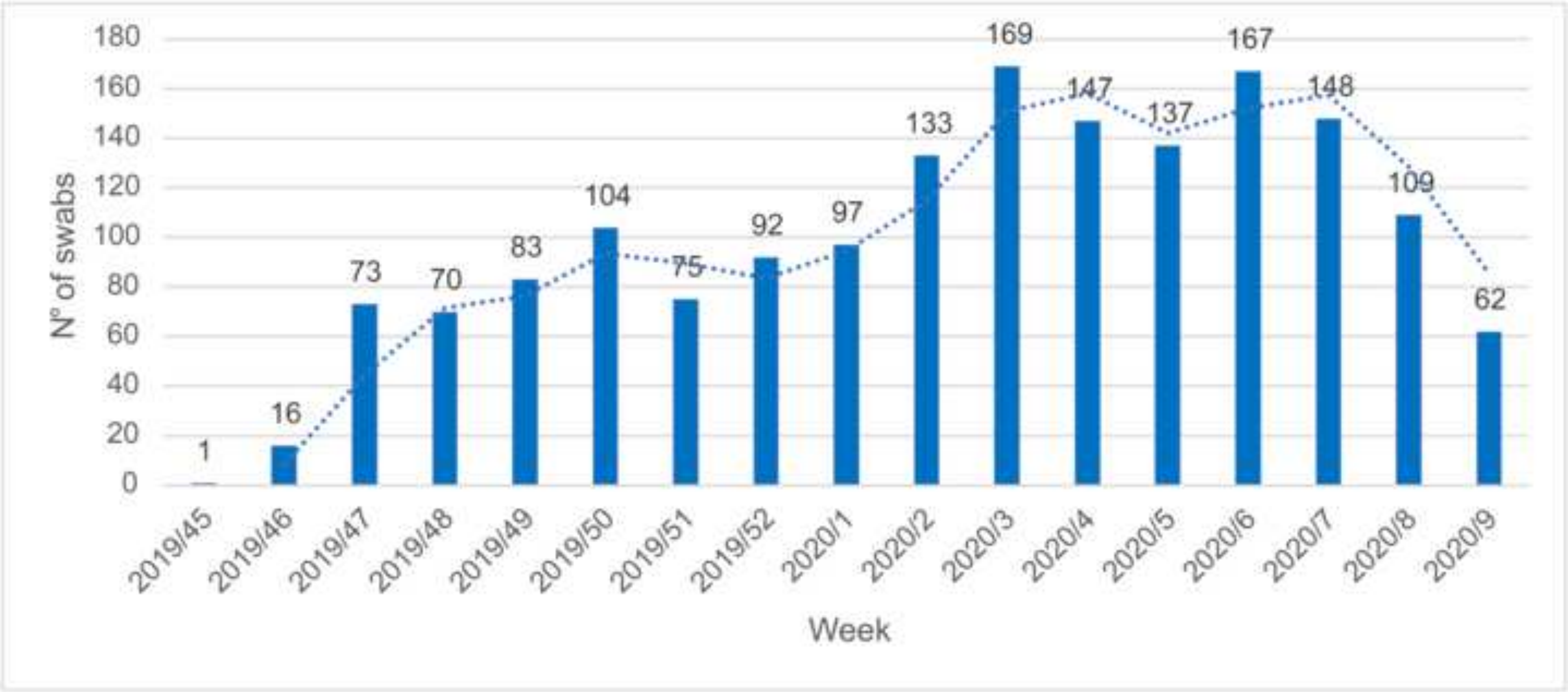

Figure 2

Click here to access/download;Figure;Fig2.tiff

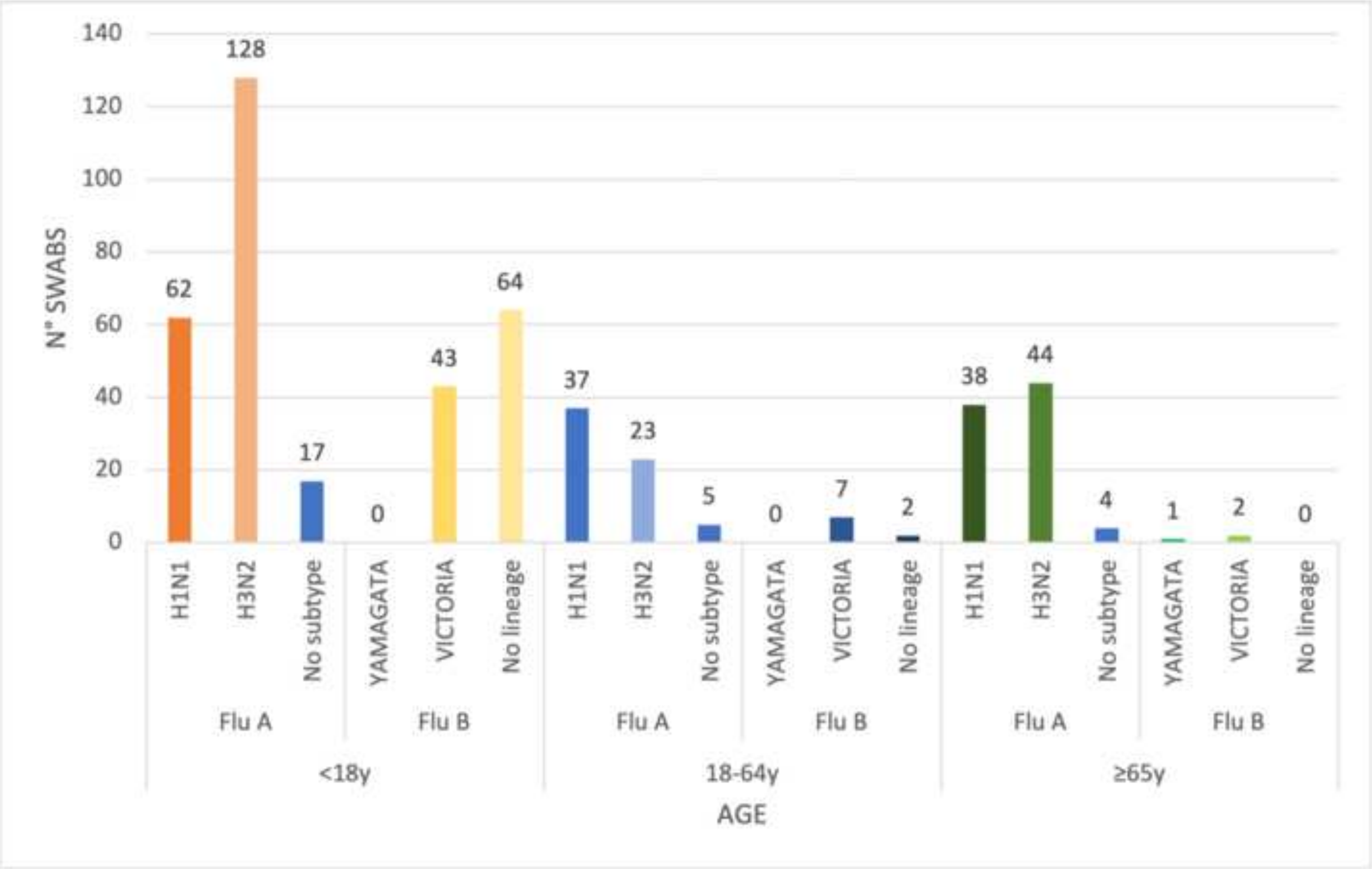

Supplement: Attachment — Submitted filename: PONE-D-21-10636_reviewer_corrected.pdf [file pone.0260947.s002.pdf]
