# Supplementary material for: No evidence of SARS-CoV-2 in hospitalized patients with severe acute respiratory syndrome in five Italian hospitals from 1st November 2019 to 29th February 2020
Source: PLoS One. 2021 Dec 7;16(12):e0260947. doi: 10.1371/journal.pone.0260947 (PMC8653811; doi:10.1371/journal.pone.0260947)
Supplement: Attachment — Submitted filename: Response_to_reviewers_PONE-D-21-10636.docx [file pone.0260947.s003.docx]

Genoa, 10 August 2021

Dear Academic Editor PLOS ONE,

Thank you for giving us the opportunity to resubmit our manuscript “No evidence of SARS-CoV-2 in hospitalized patients with severe acute respiratory syndrome in five Italian hospitals from 1^st^ November 2019 to 29^th^ February 2020” (PONE-D-21-10636) for publication in your journal. The reviewers' comments were insightful and pertinent.

Below, you will find the reviewers' reports with point-by-point replies and explanations of the modifications made. The reviewers’ comments are **in bold***,* while our replies are *in italics*.

The revised manuscript has been uploaded to the system in two versions: one with “track changes” and the other without “track changes”.

Sincerely,

Prof. Donatella Panatto

**Journal requirements**

1) The manuscript meets PLOS ONE’s style requirements.
2) The sentence “The study protocol was approved by the Ethics Committee of the Liguria Region (Genoa, Italy) (n◦ 245/2019) as coordinator center and subsequently approved by all local the Ethics Committees” has been added.
a) In the manuscript, the paragraph “Ethics Statement”, containing the EC approval code and the detail on informed consent, has been added.
3) The sentence “Informed written consent was obtained from each patient, as required by the DRIVE study protocol [15]” has been added.
4) In the Methods section, the description of the hospitals has been added.

**Reviewers’ comments**

Reviewer #1:

**All the sections of the manuscript need to be rewritten or to be rephrased with more focus on the aim of the work and the value of the results. the sections need to be better connected to show the importance of the findings. There are some drawbacks in the sampling and storing. These drawbacks should be discussed to clearly show their effects on the results and their reliability.**

*Done*

*As required by reviewer 1, all sections have been rewritten in order to better focus on the aims and results of the study.*

*All revisions are well visible in the revised manuscript with track change.*

Reviewer #2:

1) **Were the tests for all the viruses done simultaneously? The authors have to rule out the possibility that RNA in the samples was not degraded to yield a positive test for SARS-CoV-2.**

*The text has been modified in order to better explain that the molecular analyses for influenza and SARS-CoV-2 were not performed simultaneously. The molecular test for influenza was performed before the test for SARS-CoV-2. The study biases have been better explained.*

2) **Are the authors sure that the RT-PCR tests were real time?**

*Yes, all molecular tests were performed by means of the real-time method.*

3) **Which of the four cities are located in Northern Italy (the worst affected region)?**

*Genoa is the only city located in Northern Italy*

4) **ECDC? European Center for Disease Control?**

*Yes*

5) **Table 1: What does N/A mean? Explain.**

*The notation N/A (not available) has been explained*

6) **Write figure legends or at least their titles.**

*Done*

References have been updated and renumbered.

Figure 2 has been improved.
