# Supplementary material for: No evidence of SARS-CoV-2 in hospitalized patients with severe acute respiratory syndrome in five Italian hospitals from 1st November 2019 to 29th February 2020
Source: PLoS One. 2021 Dec 7;16(12):e0260947. doi: 10.1371/journal.pone.0260947 (PMC8653811; doi:10.1371/journal.pone.0260947)
Supplement: Attachment — Submitted filename: Response_to_reviewers_PONE-D-21-10636_4112021.docx [file pone.0260947.s004.docx]

Genoa, 4 November 2021

Dear Academic Editor PLOS ONE,

Thank you for giving us the opportunity to resubmit our manuscript “No evidence of SARS-CoV-2 in hospitalized patients with severe acute respiratory syndrome in five Italian hospitals from 1^st^ November 2019 to 29^th^ February 2020” (PONE-D-21-10636) for publication in your journal. The reviewers' comments were insightful and pertinent.

Below, you will find the reviewers' reports with point-by-point replies and explanations of the modifications made. The reviewers’ comments are **in bold***,* while our replies are *in italics*.

The revised manuscript has been uploaded to the system in two versions: one with “track changes” and the other without “track changes”.

Sincerely,

Prof. Donatella Panatto

**Reviewers’ comments**

Reviewer #1:

**All the comments have been addressed. I recommend the authors to rewrite the conclusion section**

*Done*

Reviewer #2:

**The authors have indicated that the detection for SARS-CoV2 was done on archived samples that were previously tested for Influenza viruses. They found that all the samples were found negative for the coronavirus. The onus is on the researchers to show that the RNA in the samples was not degraded. They should have used some positive controls for these samples. For example test again for the already detected Influenza virus. Furthermore, they should indicate whether they re-extracted RNA from the samples or used already extracted RNA from these samples. These are extremely important points. For this reason, major revision is indicated.**

*As required by reviewer 2, we have better specified, in the Materials and Methods section and Discussion section, the method of verifying the correct re-extraction of the genetic material from the sample and the integrity of the viral RNA.*
